# Older People’s Perspectives on Health, Physical Activity and Nutritional Behaviors

**DOI:** 10.15171/hpp.2015.034

**Published:** 2016-01-30

**Authors:** Leila Alizadeh, Leili Salehi

**Affiliations:** ^1^Department of Maternal and Child Health, Ardabil Branch, Islamic Azad University, Ardabil, Iran; ^2^Department of Health Education and Promotion, School of Public Health, Alborz University of Medical Sciences, Karaj, Iran

**Keywords:** Elderly, Nutritional behavior, Physical activity, Qualitative study

## Abstract

**Background: ** Approaches for investigating health-promoting lifestyle generally focus on physical activity and regular diet. To explore the perspectives of Iranian elders regarding health, healthy eating and physical activity (PA) this study was conducted in 2012.

**Methods:** Participants in this qualitative study were selected through purposeful sampling. Ten focus groups were conducted with 60 older adults in 3 elderly centers in Tehran. A moderator’s guideline that consisted of general and specific questions was used. Focus groups were audio recorded, transcribed verbatim and analysis was performed using conventional content analysis.

**Results:** Participants explained their perspectives regarding health, healthy eating and PA in the following 5 categories: meaning of health was represented based on issues such as absence of pain and disorder, complete body wellbeing, staying away from hazards, complete individual satisfaction, experiencing positive events, effective communication, faithfulness and trust in God. The healthy eating category was featured by adequate eating, age balanced diet, refraining from under or over nutrition and sensible consumption of fruits and vegetables. The PA was described - according to the level of performing outdoor activities or household tasks. Expressions about the perceived benefits and barriers of healthy eating and PA were aligned the two remaining categories.

**Conclusions:** Participants have referred to the association between both PA and dietary practices and health. Understanding how older people define physical activity and nutritional behavior and recognition of the most important perceived benefits and barriers that might contribute to have a healthy eating or adequate PA profile could procure insight into the type of interventions that are required to promote healthy lifestyle among Iranian older adults.

## Introduction

The current global growth pattern in the size of older population may pose great political, economic, and social consequences worldwide.^1^ There is a considerable interest though, in health promotion studies to examine advisability of different lifestyle change strategies in impelling positive health consequences for ageing population.^2^ Adapted approaches to promote healthy lifestyle amongst elders in the conducted studies were generally included conventional health related lifestyle practices such as doing regular exercise and having a regular diet.^3^

But despite the existing research evidence that highlight benefits of healthier lifestyle for old adults yet 70% of persons over the age of 60 years old have sedentary lifestyle and^4^ fewer than 40%  consume the recommended five serving a day of fruits and vegetables in many parts of the world.^5^ This may suggest the evidence gap that still exist in spite of the broad range of the published qualitative and quantitative studies on importance of healthy diet and physical activity among elderly people.^6,7^

Consistent with the global trend of aging, the proportion of old population relative to the whole population has also increased over the last few decades in Iran.^8^ According to the estimations of the United Nations Statistics Division, proportion of the elderly population in Iran will increase from 5.4% in 1975 to 10.5% and 21.7% in 2025 and 2050 respectively.^9^ But not withstanding with this growing size of the country’s old population, the number of studies to explore influencing factors of healthy behaviors such as daily consumption of fruits and vegetables or performing regular physical activities is meager. To persuade healthy lifestyles amongst the Iranian older population, development of evidence based interventional programs that are fitted to the socio-cultural context of the community is a matter of the utmost importance.^10^

The aim of this study was to explore the perspectives of Iranian elders regarding health, healthy eating and physical activity to inform future research and target settings in the health planning.

## Materials and Methods

### Design

The present study employed a qualitative approach (Phenomenology) for data collection and analysis in order to describe the health, healthy eating and physical activity through the Iranian elders’ points of view.

### Participants & Procedures

Those elder people who have been registered as a member of Tehran’s three elderly centers were recruited into the study through the purposeful sampling method in 2012. Convenience sampling was used to choose the three elderly centers (Jahandidaghan, Mehravar, Omid) amongst the 23 day care centers in Tehran. In these centers free educational and social services are provided to the eligible members i.e. those aged 60 years old or above. Inclusion criteria to include the study participants were a list of predetermined characteristics including age 60 years or over, being a registered member in the selected elderly centers and not having history of hospitalization in the past four weeks.

### Data collection

To collect the study data ten focus groups, each composed of six participants of elderly people to allow engaging conversation were conducted at elderly centers in Tehran. Each focus group in the study was scheduled for 1.5-2 hours and continued until data saturation.

A moderator’s guideline was prepared consisting of general and specific questions that were supplemented by probes to ensure smooth conduction of the sessions. These questions were formulated in consultation with specialists (Box 1). A moderator managed each session and an assistant took hand written notes of the discussions to be used for data analysis purposes. Before beginning each session formally, demographic characteristics of the participants were collected‏.

The sessions started with an introduction and general outline of the interview questions. Each participant was requested to present her/his life story in approximately 5 minutes. The involvement and participants’ interactions were encouraged throughout all sessions in order to explore the participants’ perspectives regarding their general health. The focus group discussions were also tape recorded and then transcribed verbatim in Persian and subsequently the transcripts were translated into English by the lead author. The focus group transcripts underwent basic content analysis; data were open coded by the researchers, similar codes were grouped into categories then were aggregated into four themes.

### Research ethics

Permission to conduct this study was obtained from the Ethics Committee of Tehran University of Medical Sciences, Tehran, Iran‏. The researchers also obtained the participants’ permission to audiotape each interview. All the participants were informed about the purpose of the study and if any participant was disagreed to take part in the study; he/she was excluded. Therefore, informed consent sheet was signed by all of the participants prior to the study entrance.

### Trustworthiness

To unsure trustworthiness and in particular credibility of the data collected, same moderator was appointed for all focus group discussion sessions and cross- checking and analysis of the study data were performed collaboratively.

## Results

A total of 60 participants took part in this study. Their age range was from 60 to 97 years old with an average of 65.77 (SD 4.98) years. The sample consisted of 34 females (56.66%). Table 1 shows the demographic profile of the participants.

**Table1:** Demographic profile of the participants in the 10 conducted focus groups

Age groups (yrs)	Education	Focus group (n)	Female (n)	Male (n)
60-64	Illiterate	3	8	10
60-64	literate	2	6	6
65-69	Illiterate	1	4	2
65-69	literate	1	4	2
70-74	Illiterate	2	8	4
≥75	Illiterate	1	4	2

The participants came from different socioeconomic background. All of them were married, and only 30% (N = 18) were literate. In terms of nationality, all of the participants were Iranian and could spoke Persian as the country’s formal first language. Nearly all of them suffered from a kind of chronic disease especially musculoskeletal pain.

Four main categories emerged based on the data analysis and several distinctive subcategories were identified within each category. These categories and their subcategories represent the main factors influencing the Iranian elders' perspectives on health, healthy eating and physical activity. The categories were meaning of health‏, healthy eating and regular PA and the factors associated with the perceived benefits and barriers of healthy eating behavior and PA. These categories and their subcategories were shown in Table 1.

### Meaning of health and its influencing factors 

One of the main categories that emerged from the data analysis was “meaning of health” with several subcategories according to the participants’ experiences.

“The health is complete body wellbeing, complete individual satisfaction and absence of pain and disorder”. (An e*lderly man, 72 years old, illiterate*)

Most of the participants believed that health is physical and mental wellbeing. Majority of them explained that healthy eating and regular physical activity are essential for physical wellbeing.

“Being able to communicate effectively and intimately with others was essential for social wellbeing”. (An *elderly women, 69 years old, illiterate*)

“As my father in village always practice healthy eating and works hard, he has a good physical health in spite of his old age I understand that for being healthy, I should consume healthy foods and be active”. (An *elderly woman, 60 years old, illiterate*)

“I believe having a strong faith is essential for spiritual wellbeing”. (An *elderly man, 74 years old, illiterate*)

The majority of participants believed that different physical and social factors such as trusting in God, continuous and desirable level of activity, good cultural and familial habits, developing healthy habits early in life, desirable social growth, living in present time and not thinking about past or being worry about the future, having a social support, refraining from hazardous activities, traveling, helping others, experiencing positive events throughout the life, reducing anxiety and sleeping with light food in night could influence physical, mental, spiritual and social wellbeing.

One elderly man said:* "*Eat your breakfast alone, lunch with your friends and dinner with your enemies". (A *66 years old, literate respondent*)

Meaning of this sentence was that you must eat your breakfast complete while concentrated, your lunch in moderate but your dinner in least.

The findings of this study indicated that the elder people’s religious beliefs have had impact on their health attainment process and increased the likelihood of adhering to an improved recommended life style.

Furthermore, the study participants’ religious beliefs were indicative of the boosting effects it may have on self-confidence of the recruited old people in doing recommended activities.

One of the women told: 'If God wills, we will be able to do everything, If God wants we will live longer and stay healthy". (A 60* years old, illiterate respondent*)

### Perspectives on healthy eating and physical activity 

Nearly most of the participants revealed that healthy eating means adequate eating and avoidance from under or over nutrition, having a balanced diet according to the age and consumption of recommended daily allowance of fruits and vegetables.

“I believe healthy eating means that you should have enough food, no more and no less. If you eat more you will be ill and if you eat less, you don't have energy to work”. (An *elderly man, 65 years old, illiterate*)

“We are not young, we are not able to eat everything for example those foods that are not good for our stomach. I have blood pressure and healthy eating means eating according to the age requirements”. (An *elderly woman, 73 years old, illiterate*)

“Vegetables and fruits are an important part of a healthy diet, when we were child we were going to garden and eating lots of fruits from trees. That time almost all people were healthy and there was not any cancer, diabetes and blood pressure. But these days since we eat less fruits we are all ill”. (An *elderly man 76 years old, illiterate*)

According to the participants’ points of view in this study healthy eating behavior could be influenced by age, level of activity, income and mass media programs or advertisements.

One of the participants stated that” healthy eating is a really big issue for older people since, almost all are sick. Those who have high blood pressure should avoid salty foods, those with diabetes should avoid sweet foods, but when you are young you may eat any things”. (An e*lder woman, 72 years old, illiterate*)

“It is more expensive to buy healthy foods than a fast food. Unhealthy foods are cheaper than healthy foods”. (An *elderly woman, 69 years old, literate*)

“When you are working, you must eat much more than usual to get enough energy to do your duties better”. (An *elderly man, 65 years old, illiterate*)

Mothers’ role in following healthy diet was another important raised factor to have a familial healthy nutrition because in Iran, mostly mothers prepare food for the family members.

One of the participants explained that* "*Mothers have the important roles in directing their other family members to consume healthy foods*".* (An *elderly man, 64 years old, illiterate*)

When the study participants were asked about their preparedness in following a healthy eating program they referred to their appropriate level of self-efficacy in pursuing the recommendations. Almost all of the participants (except one) verified that they would be able to follow a healthy nutritional guideline if they were given an efficient diet program.

"If I personally believe in the efficacy of a nutritional program, I will follow that diet even if my family members oppose it*".* (An *elderly woman, 65 years old, literate*)

The participants in this study believed that health care providers and mass media programs could have key role in familiarizing people with healthy eating behaviors.

"While several advertisements are broadcasted by the television channels about chips or chocolates all the time, how we can expect community members to have a healthy diet*".* (An *elderly woman, 66 years old, illiterate*)

According to the results of this study, recreational, outdoor activities and housework were the main emerged themes to describe physical activities. Most of the interviewees declared that no difference exists between a physical activity and an exercise. About 50% of the participants declared that housework is a kind of physical activity.

“I work all the day in kitchen, washing, cooking, caring from grandchildren, all these works for me are physical activities, so I don’t need to exercise”. (An e*lderly woman, 61Years old, literate*)

Nearly all participants stated that if they decide they will be able to exercise regularly 3 times a week for at least 20 to 30 minutes in each session.

### Perceived benefits and barriers to healthy eating behavior

Participants in the present study indicated that benefits of healthy eating might be whole body health, disease prevention and weight control.

“When we are ill, the doctors recommend us to eat fruit and vegetables much more”. (An *elderly woman, 69 years old, illiterate*)

“You avoid obesity when you eat healthy food and avoid from excess intakes” (An *elderly women, 73 years old, illiterate*)

According to the study respondents’ points of view socio-cultural factors were most important influential factors in having a healthy eating behavior.

The main barriers of having healthy diet were also discussed in the groups. The majority of the participants stated that factors such as family’s customs and traditional food preferences which were mainly toward carbohydrate and fat consumption, cost of eating healthy foods, mothers’ role in selecting an unhealthy diet, and desire for fast food eating due to their taste were barriers to engage in healthy eating behavior.

“Changing family’s customs is difficult, when accustomed to eat fatty meal in family, and traditional foods, how can I avoid unhealthy meals”. (An e*lderly man, 63 years old, literate*)

“Kids of the current generation like eating fast foods as our children and grandchildren do so. It is inevitable therefore for us to eat these kinds of foods”. (An *elderly man, 64 years old, literate*)

Low income was also introduced as a major barrier to include sufficient amounts of fruits and vegetables in the diet.

"Lack of enough money may restrict me from buying fruits and vegetables or other healthy foods*". (An elderly man, 70 years old, illiterate)*

Elder people’s health related limitations to consume fruit and vegetable were indicated to be another type of barrier which was reflected in the following sentence:

"When I don't have teeth, how I can eat fruit and vegetable"? (*An elderly man, 72 years old, illiterate*)

Additionally, the participants stated that meats and fruits are not qualitatively same as those they have consuming before. They explained that the foods are artificial these days and so they do not taste like previously.

“These days, everything is artificial, the tastes of food differ from the past times, yesterday I bought cucumber, but it was tasteless like water”. (An *elderly man, 79 years old, illiterate*)

### Perceived benefits and barriers to physical activity

Almost all of the participants stated that physical activity was beneficial for their health. “Exercise is good for health; it’s good, if you exercise. As long as you exercise, you’ll be healthy”. (An *elderly woman, 76 years old, illiterate*)

A majority of the study participants were in agreement that regular physical activity may have benefits such as meeting a lot of people and communication with them, decreasing of tensions, being open minded, feeling a higher level of endurance, hopefulness, better sleeping, flexibility and adaptability with difficulties, decrease in Alzheimer’s sign, improvement of health, improvement of body functions such as cardiovascular or respiratory systems output, prevention of obesity and increase of body strengths that all achievable through rigorous physical activity. The study respondents were in believe that by overthrowing of the perceived barriers to do physical activity they may have better physical activity profile.

“To be active means being open minded, feeling courage higher level of endurance, hopefulness, having better sleep times, a good coping ability in dealing with difficulties, decrease of Alzheimer’s sign and in total improvement of health”. (An e*lderly women, 62 years old, literate*)

“Exercise helps me to get rid of disease and improve functions of my body systems like my heart and blood flow in my arteries”. (An *elderly man, literate, 63 years old*)

“I used to take a walk outdoor every day. By doing so I could contact my friends and meet a lot of people”. (An elderly man, 64 years old, literate)

Participants identified that group based exercises are an important source to encourage people to have physical activity.

“I do not like to exercise alone, after every session of a group based exercise, I feel good sense. I feel that I like to continue and exercise much more”. (An *elderly man, 64years old, literate*)

A large number of participants believed that regular physical activity did not need specific facilities and so everyone can do it in any situation.

In terms of barriers to physical activity, participants raised issues that could be grouped under 3 themes: 1) Lack of motivation and have a gloomy mood 2) physical health limitations and 3) cultural and custom related barriers.

Many participants referred to lack of motivation as a major barrier.

They spoke about factors like feeling lazy, eating a lot, air pollution, depression, fear of accident and injury, lake of an accompanying person, high tempered weather and religious barriers that might make them unwilling and unmotivated to engage in a regular physical activity.

“Everyone knows that exercise is good for health but laziness don’t allow me to exercise, we need someone who encourage us, we are not self-motivated to do exercise”. (An *elderly women, 63 years old, literate*)

“Eating a lot food doesn’t allow someone to move, after meal I do like to sleep”. (An *elderly man, 63 years old, illiterate*)

“In hot and polluted weather, all of us are lethargic and unwilling to be physically active”. (An e*lderly woman, 77 years old, illiterate*)

“Few days ago my friend came out of her house for walking; a car crashed her and escaped. I didn’t feel safe anymore when walking”. (An *elderly woman, 66 years old, illiterate*)

Living in small apartment was also mentioned as a kind of barrier for exercise, a number of the study participants stated that small space of their apartments does not allow them to do physical activity.

“When we live in 45 or 50 meter apartments, how we can exercise, out of our house, air is polluted, there is not any suitable space to move and exercise. The only things we can do is sitting down and complaining”. (An e*lderly woman, 63 years old, illiterate*)

Wearing scarf that is a part of recommended code of dressing for women was mentioned as a kind of barrier just by one of the participated women.

“How can I exercise with scarf, we (Muslim women) are wearing scarf, it isn’t allow us to move and to have fun (An *elderly woman, literate, 60 years old*)”

## Discussion

This study carried out in order to investigate the perspectives of Iranian elderly people about healthy lifestyle. Findings showed that the participants’ perspectives regarding health and its influencing factors are positive. The majority of the participants referred to all physical, mental, social and spiritual dimensions of health. Furthermore, they explained the role of healthy eating in decreasing morbidity rates, increasing life expectancy and quality of life. However, according to the participants’ points of view healthy nutritional habits are established from childhood. The participants also were in believe that cultural nutrition habits could form primary eating behaviors in children and it will continue throughout the life. Based on the participants’ viewpoints, in the Iranian culture, mothers are responsible to take care of their children and to prepare foods for all their family members including their children. So their beliefs regarding the suitable preferred foods could influence establishment of healthy eating behaviors.

These findings are consistent with the results of other studies in which the role of women’s perspectives on promoting healthy behaviors has been showed.^11,12^

A significant finding of this study related to religiosity of Iranian older people, their belief in God and their confidence that if God will, everything including their health would be reserved. All the study participants in this study were faithful and were in believe that they would be able to manage having a regular healthy diet or doing regular exercise if they rely on God.

The study participants also referred to the socio-economic factors including traditions which encourage certain eating behaviors that are common in the Iranian families and could shape up eating habits especially among illiterate individuals. This study showed the unhealthy eating habits in most cases are actually rooted in the Iranian traditions for instance serving some especial foods in the familial ceremonies which are unhealthy but favorable for Iranian tastes. One of the important barriers reported in this study was economic barrier to buy fruits and vegetables that is consistent with the findings of other studies.^13,14^ Further research to understand the role of different cultural and socio- economic factors on eating habits of Iranian elders is recommended.

Physical health limitations were explained as one of the most important barriers to do regular exercises by the participants which is concurrent with the findings of another study.^15^ The participants stated that overcoming the perceived barriers to do regular physical activity could increase probability of engagement in a scheduled physical activity plan.

The results of the present study showed that advertisements in mass media especially television could have important role in promoting healthy life style and establishing people’s habits and preferences. Such a finding is consistent with the results of a previously conducted study.^16^ According to the participants' viewpoints mass media along with social support from friends, family members and health care providers play an important role in acquiring healthy behaviors. Therefore, broadcasting educational messages and programs about healthy lifestyle related issues through mass media to create or boost enthusiasm towards healthy behaviors among Iranian elder adults is strongly recommended. To be effective mass media programs should be attractive, clear, and culturally appropriate and also be compatible with the audiences’ age^17^ and the stages of change that are intended.^18^

Role of the formal and informal social support to develop healthy behaviors among elderly people was also addressed in the studies.^19^It is suggested in the conducted studies that provision of positive social support could be an effective method in enhancing self-care behaviors.^19^

Based on the findings of the current study religious beliefs amongst older people could also play a major role in enhancing self-efficacy and therefore; going after a healthy lifestyle. It could be important to highlight the role of religious beliefs on health promotion activities when designing educational programs for elderly population of Iran. Such a recommendation was also mentioned in another study.^19^

This study results indicated that for the recruited elderly people of Tehran barriers to have regular exercise is much lesser than the perceived barriers to have a healthy eating behavioral pattern. The limitations for women in doing physical activities as one of the study respondents declared are more likely to exist compared to men due to the dressing codes women should comply in public places. This limitation however; could be removed by structuring appropriate and convenient special spaces for women that encourage them to engage in regular physical activities.

## Conclusion

The beliefs, values, and customs of Iranian elderly people regarding healthy eating and physical activity would establish their health related behaviors. All these cultural factors affect individuals from childhood until their later life. Therefore, in developing strategies of health education and promotion for acquisition of a healthy lifestyle by elder people including healthy eating behavior or regular physical activity socio-cultural characteristics, normative beliefs and values with cross-generation influences throughout the entire lifespan must be considered carefully.

## Acknowledgements

We are grateful to all the participants.

## Conflict of interest

All authors declare that they have no competing interest.

## Appendix

**Appendix 1:** Interview schedule for focus groups

1. Describe the meaning of health?
2. Which factors could lead to health and wellbeing?
3. What is the meaning of healthy eating?
4. Some people are not eating healthy. Can you tell me some of the reasons why you and other older persons eat healthy? How about physical activity?
5. What is the difference between exercise and physical activity?
6. Who would be a good source of encouragement and support for older people to participate in physical activities or practice healthy eating?
7. Is there anything else about healthy eating or physical activity that we haven't discussed about and you think it is important?
8. How certain are you that you can have healthy eating?
9. How certain are you about doing regular physical activity?
